# Clinical Outcomes of Hospitalization for Severe Hyperkalemia in the Absence of Life-threatening Manifestations

**DOI:** 10.31662/jmaj.2026-0089

**Published:** 2026-08-21

**Authors:** Shinnosuke Sugihara, Takashin Nakayama, Tatsuhiko Azegami, Motoaki Komatsu, Kaori Hayashi

**Affiliations:** 1 Division of Nephrology, Endocrinology, and Metabolism, Department of Internal Medicine, Keio University School of Medicine, Tokyo, Japan; 2 Division of Nephrology, Department of Internal Medicine, Tokyo Saiseikai Central Hospital, Tokyo, Japan

**Keywords:** hospitalization, hyperkalemia, mortality, outpatient management, recurrence

## Abstract

**Introduction:**

Severe hyperkalemia warrants immediate hospitalization for patients with acute instability. However, the benefit of inpatient care in the absence of acute instability remains poorly defined. We therefore evaluated clinical outcomes across clinical settings for these patients.

**Methods:**

This two-center retrospective cohort study included outpatients presenting with severe hyperkalemia (serum potassium level [K^+^] ≥6.5 mEq/L). Among the initial cohort, our primary analysis focused on patients without life-threatening arrhythmias or other concurrent acute complications; the risk of 90-day all-cause mortality and hyperkalemia recurrence was compared between inpatient and outpatient management using overlap weighting.

**Results:**

A total of 554 patients with severe hyperkalemia were identified, comprising 276 inpatients and 278 outpatients. Median age was 75 (interquartile range [IQR] 65-83) years, 392 (71%) were male, and median K^+^ was 6.7 (IQR 6.5-7.0) mEq/L. In the analysis of 374 patients whose presentation was stable aside from the K^+^ elevation itself (99 inpatients and 275 outpatients), weighted logistic regression showed no significant difference in 90-day all-cause mortality between the two groups (odds ratio [OR] 1.39, 95% confidence interval [CI] 0.31-6.26); no hyperkalemia-specific deaths occurred. In contrast, outpatient management was associated with a significantly higher risk of hyperkalemia recurrence (OR 4.82, 95% CI 1.04-22.44).

**Conclusions:**

While outpatient management for severe hyperkalemia might be feasible for clinically stable patients in terms of short-term mortality, it carries a substantially increased risk of recurrence. Accordingly, hospitalization should generally be regarded as the preferred strategy.

## Introduction

Hyperkalemia is a potentially life-threatening electrolyte disturbance, largely due to its association with fatal cardiac arrhythmias ^(1), (2)^. It is highly prevalent among patients with chronic kidney disease (CKD), heart failure, and diabetes mellitus, and its clinical relevance is expected to increase further with the expanding use of medications that disrupt potassium homeostasis, including renin–angiotensin system (RAS) inhibitors and mineralocorticoid receptor antagonists (MRAs) ^(3), (4)^.

Detailed guidance is available regarding the diagnostic workup and medication management of hyperkalemia ^(5), (6)^. However, evidence to guide hospitalization decisions remains scarce ^(7)^; the Kidney Disease: Improving Global Outcomes conference report does not provide explicit criteria for hospital admission ^(5)^. While hospitalization is clearly warranted in the presence of electrocardiographic (ECG) abnormalities or concurrent acute illness, it remains uncertain whether inpatient care confers significant benefits among clinically stable patients with hyperkalemia. Given the potential harms associated with hospitalization as well as the burden on the healthcare system, this represents an important research question ^(8), (9), (10)^.

Therefore, this study sought to address clinical uncertainty by comparing outcomes between patients with severe hyperkalemia managed as inpatients and those managed as outpatients. By providing evidence in an area that has relied largely on subjective clinical judgment, these findings may help guide physician decision-making and improve the quality of care.

## Materials and Methods

### Study participants

This retrospective cohort study was conducted at Keio University Hospital and Saiseikai Central Hospital in Japan. We identified patients aged ≥18 years who presented to the outpatient department between March 2014 and July 2024 with serum potassium level (K^+^) ≥6.5 mEq/L. For patients who experienced multiple episodes of hyperkalemia during the study period, only the first event was included in the analysis. We excluded patients who (1) were diagnosed with pseudohyperkalemia, (2) were suspected to have hyperkalemia due to iatrogenic fluid contamination, (3) were in cardiopulmonary arrest (CPA) upon arrival, (4) were receiving maintenance hemodialysis or peritoneal dialysis, (5) had missing data for variables measured either prior to or at the time of the index event (precluding accurate assessment of concurrent acute conditions), or (6) were transferred to another healthcare facility. The decision regarding inpatient versus outpatient management was not guided by predefined institutional criteria but was determined at the discretion of the treating physicians in routine clinical practice.

The study protocol was approved by the Ethics Committee of Keio University School of Medicine (approval number: 20251164), and informed consent was obtained via an opt-out approach provided on the institutional website.

### Data collection

Clinical information was retrospectively collected from the electronic medical records of the participating hospitals. Data at the time of the hyperkalemic event included demographics (age, sex, and body mass index [BMI]), comorbidities (diabetes mellitus, heart failure, coronary artery disease, stroke, and malignancy), medication use (RAS inhibitors, MRAs, beta-blockers, loop diuretics, bicarbonate, and potassium binders), visit pattern (scheduled or unscheduled), presence of life-threatening arrhythmias (complete atrioventricular block or ventricular tachycardia) or concurrent acute conditions (infection, acute heart failure, acute coronary syndrome, gastrointestinal bleeding, diabetic ketoacidosis, or others), do-not-resuscitate (DNR) status, and management strategies for hyperkalemia. Laboratory data were obtained for K^+^ and creatinine at the time of hyperkalemia and 3 months prior, as well as complete blood count (CBC) at the time of the event. Estimated glomerular filtration rate (eGFR) was calculated using the Japanese equation: eGFR = 194 × creatinine^-1^·^094^ × age^-0^·^287^ (× 0.739 if female) ^(11)^.

### Outcomes and definitions

The primary outcomes were all-cause mortality and recurrent hyperkalemia within 90 days of the index event among patients without life-threatening ECG abnormalities or concurrent acute conditions. Causes of death were additionally assessed and categorized as non–hyperkalemia-specific (infection, malignancy, cardiovascular disease, or others) or as hyperkalemia-specific mortality (defined as death due to fatal cardiac arrhythmias).

### Statistical analysis 

Continuous variables were expressed as medians with interquartile ranges (IQRs) and categorical variables as counts and percentages. We applied propensity score–based overlap weighting to achieve covariate balance between the inpatient and outpatient groups. Propensity scores were estimated using a logistic regression model that included age, sex, BMI, comorbidities (diabetes mellitus, heart failure, coronary artery disease, stroke, and malignancy), medication use (RAS inhibitors, MRAs, beta-blockers, loop diuretics, bicarbonate, and potassium binders), visit pattern, DNR status, laboratory values at the index event (K^+^, creatinine, white blood cell count, hemoglobin level, and platelet count), change in K^+^ over the preceding 3 months, and creatinine ratio (the value at the index event divided by that measured 3 months earlier). Covariate balance between the groups before and after weighting was assessed using standardized mean differences (SMDs), with values <0.1 considered to indicate a negligible imbalance. Weighted logistic regression models were used to compare 90-day outcomes between the two groups.

All statistical analyses were performed using Stata version 18.0 (StataCorp LLC, College Station, TX, USA). A two-sided p-value <0.05 was considered statistically significant.

## Results

### Patient characteristics

Among 2,452 outpatients with K^+^ ≥6.5 mEq/L who were screened, 554 were included in this study (Figure 1). Of these, 276 (50%) were managed as inpatients and 278 (50%) as outpatients. Baseline characteristics of the study participants are detailed in Table 1. Compared with the outpatient group, the inpatient group had a higher proportion of patients with DNR status (29% vs. 5%; absolute SMD = 0.68) and unscheduled visits (72% vs. 2%; absolute SMD = 2.10). Concurrent acute conditions were rarely observed in the outpatient group, occurring in only three patients; no life-threatening ECG abnormalities were identified in this group. Regarding comorbidities, malignancy was more prevalent among inpatients than outpatients (53% vs. 35%; absolute SMD = 0.36), whereas diabetes mellitus was more common in the outpatient group (57% vs. 41%; absolute SMD = 0.34). In addition, the inpatient group was less likely to receive RAS inhibitors (49% vs. 74%; absolute SMD = 0.54), MRAs (19% vs. 24%; absolute SMD = 0.11), bicarbonate (5% vs. 12%; absolute SMD = 0.24), or potassium binders (9% vs. 22%; absolute SMD = 0.37). The inpatient group had higher K^+^ (6.8 vs. 6.6 mEq/L; absolute SMD = 0.71) and creatinine levels (2.7 vs. 2.5 mg/dL; absolute SMD = 0.23), as well as a greater 3-month change in K^+^ (2.2 vs. 1.2 mEq/L; absolute SMD = 1.14) and a higher creatinine ratio (1.8 vs. 1.1; absolute SMD = 0.54), than outpatients. Baseline characteristics of the primary analytic cohort (99 inpatients and 275 outpatients) are presented in Supplemental Table S1. After applying overlap weighting, adequate covariate balance was achieved between the two groups, with all absolute SMDs below 0.1.

**Figure 1.**
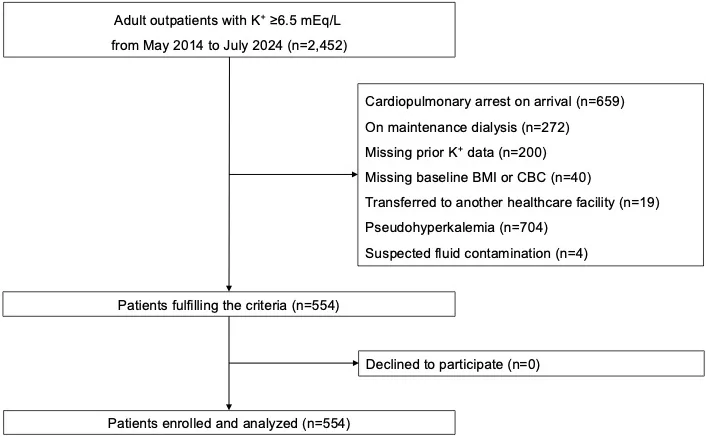
Flow diagram of patient selection. A total of 2,452 adult outpatients with K^+^ ≥6.5 mEq/L were screened between May 2014 and July 2024. Of these, 1,898 patients were excluded based on the following criteria: pseudohyperkalemia (n = 704), cardiopulmonary arrest on arrival (n = 659), being on maintenance dialysis (n = 272), missing prior potassium data (n = 200), missing baseline BMI or CBC (n = 40), transfer to another healthcare facility (n = 19), and suspected fluid contamination (n = 4). After applying all exclusion criteria, 554 eligible patients were enrolled in the final analysis. BMI: body mass index; CBC: complete blood count; K^+^: serum potassium level.

**Table 1.** Characteristics of Patients with Severe Hyperkalemia.

	Overall (n = 554)	Inpatient (n = 276)	Outpatient (n = 278)	Absolute SMD
Age (years), median (IQR)	75 (65-83)	75 (65-84)	74 (65-82)	0.06
Sex: male, n (%)	392 (71)	181 (66)	211 (76)	0.23
BMI (kg/m^2^), median (IQR)	22.3 (19.4-25.4)	21.0 (18.5-24.2)	23.4 (20.8-26.1)	0.16
Do-not-resuscitate code, n (%)	95 (17)	81 (29)	14 (5)	0.68
Visit pattern, n (%)				
Scheduled	351 (63)	78 (28)	273 (98)	2.10
Unscheduled	203 (37)	198 (72)	5 (2)	
Concurrent acute conditions, n (%)				
Infection	64 (12)	63 (23)	1 (0.4)	0.75
Acute heart failure	37 (7)	37 (13)	0 (0)	0.56
Acute coronary syndrome	6 (1)	6 (2)	0 (0)	0.21
Gastrointestinal bleeding	19 (3)	19 (7)	0 (0)	0.38
Diabetic ketoacidosis	18 (3)	18 (7)	0 (0)	0.37
Others	70 (13)	68 (25)	2 (1)	0.77
Life-threatening ECG abnormalities, n (%)	14 (3)	14 (5)	0 (0)	0.33
Comorbidities, n (%)				
Diabetes mellitus	271 (49)	112 (41)	159 (57)	0.34
Heart failure	200 (36)	101 (37)	99 (36)	0.02
Coronary artery disease	114 (21)	53 (19)	61 (22)	0.07
Stroke	79 (14)	38 (14)	41 (15)	0.03
Malignancy	242 (44)	145 (53)	97 (35)	0.36
Concomitant medications, n (%)				
RAS inhibitors	341 (62)	135 (49)	206 (74)	0.54
MRA	119 (21)	53 (19)	66 (24)	0.11
Beta-blockers	149 (27)	70 (25)	79 (28)	0.07
Loop diuretics	156 (28)	77 (28)	79 (28)	0.01
Bicarbonate	49 (9)	15 (5)	34 (12)	0.24
Potassium binders	84 (15)	24 (9)	60 (22)	0.37
Laboratory values, median (IQR)				
At hyperkalemia				
K^+^ (mEq/L)	6.7 (6.5-7.0)	6.8 (6.6-7.2)	6.6 (6.5-6.8)	0.71
Creatinine (mg/dL)	2.6 (1.7-4.2)	2.7 (1.8-4.5)	2.5 (1.7-4.2)	0.23
eGFR (mL/min/1.73 m^2^)	18 (11-29)	16 (11-27)	20 (12-30)	0.11
WBC (×10^9^/L)	7.2 (5.7-9.8)	8.9 (6.7-12.6)	6.5 (5.2-7.8)	0.37
Hemoglobin (g/dL)	10.7 (9.1-12.2)	10.4 (8.7-12.2)	10.9 (9.8-12.3)	0.23
Platelet (×10^9^/L)	202 (153-272)	210 (153-299)	195 (154-244)	0.30
3-month increase in K^+^ (mEq/L)	1.6 (1.1-2.3)	2.2 (1.6-2.7)	1.2 (0.9-1.7)	1.14
Creatinine ratio (hyperkalemia / 3 months prior)	1.2 (1.1-1.9)	1.8 (1.3-2.6)	1.1 (1.0-1.2)	0.54

BMI: body mass index; ECG: electrocardiographic; eGFR: estimated glomerular filtration rate; IQR: interquartile range; K^+^: serum potassium level; MRA: mineralocorticoid receptor antagonist; RAS: renin–angiotensin system; SMD: standardized mean difference; WBC: white blood cell count.

### Management strategies and clinical consequences

Details of management strategies for the inpatient and outpatient groups are presented in Figure 2. Immediate therapeutic interventions were administered almost exclusively to inpatients, including intravenous calcium (56% vs. 2%), glucose–insulin therapy (76% vs. 5%), and emergency dialysis (14% vs. 0%). With respect to oral medication adjustment, initiation or up-titration of potassium binders was more frequent among outpatients (60% vs. 43%), whereas down-titration of RAS inhibitors (43% vs. 22%) and MRAs (19% vs. 13%) occurred more often in inpatients.

**Figure 2.**
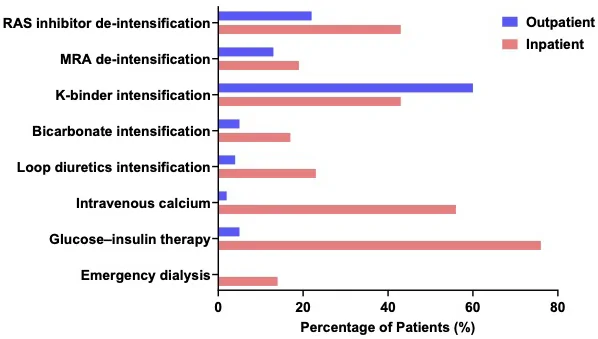
Management strategies for hyperkalemia. Deintensification is defined as reduction or discontinuation and intensification as initiation or up-titration. MRA: mineralocorticoid receptor antagonist; RAS: renin–angiotensin system.

The overall all-cause mortality was 14%, with a significantly higher incidence among inpatients compared to outpatients (27% vs. 2%) (Table 2). Malignancy (9%) was the leading cause of death, followed by infection (3%), others (2%), and cardiovascular disease (1%). No deaths directly attributable to hyperkalemia were observed in either group. Recurrent severe hyperkalemia occurred in 7% of the total cohort and was more prevalent in the outpatient setting (10% vs. 3%).

**Table 2.** Clinical Outcomes.

	Overall (n = 554), n (%)	Inpatient (n = 276), n (%)	Outpatient (n = 278), n (%)
All-cause mortality	80 (14)	74 (27)	6 (2)
Non–hyperkalemia-specific mortality	80 (14)	74 (27)	6 (2)
Infection	15 (3)	15 (5)	0 (0)
Malignancy	49 (9)	46 (17)	3 (1)
Cardiovascular disease	3 (1)	3 (1)	0 (0)
Others	13 (2)	10 (4)	3 (1)
Hyperkalemia-specific mortality	0 (0)	0 (0)	0 (0)
Hyperkalemia recurrence	37 (7)	9 (3)	28 (10)

### Comparative impact of inpatient and outpatient management

In the primary analytic cohort, all-cause mortality occurred in six inpatients and five outpatients (Table 3). Unweighted logistic regression suggested lower mortality among outpatients (odds ratio [OR] 0.29, 95% confidence interval [CI] 0.09-0.96); however, after overlap weighting, no significant difference between the groups was observed (OR 1.39, 95% CI 0.31-6.26). In contrast, there were four recurrent hyperkalemia events in inpatients and 27 in outpatients. In unweighted logistic regression, outpatient management was associated with a higher risk of recurrence (OR 2.59, 95% CI 0.88-7.59), and this association remained significant in overlap-weighted analyses (OR 4.82, 95% CI 1.04-22.44).

**Table 3.** Clinical Outcomes of Inpatient versus Outpatient Management for Severe Hyperkalemia without Concurrent Acute Conditions or Life-Threatening ECG Abnormalities: before and after Overlap Weighting.

	Setting	Participants, n	Events, n	Unweighted OR, 95% CI	Weighted OR, 95% CI
All-cause mortality	Inpatient	99	6	1 [reference]	1 [reference]
	Outpatient	275	5	0.29 (0.09-0.96)	1.39 (0.31-6.26)
Hyperkalemia recurrence	Inpatient	99	4	1 [reference]	1 [reference]
	Outpatient	275	27	2.59 (0.88-7.59)	4.82 (1.04-22.44)

Notes: Weighted estimates were obtained using propensity score–based overlap weighting, adjusted for age, sex, body mass index, diabetes mellitus, heart failure, coronary artery disease, stroke, malignancy, visit pattern, do-not-resuscitate code, use of renin–angiotensin system inhibitors, mineralocorticoid receptor antagonists, beta-blockers, loop diuretics, bicarbonate, and potassium binders, as well as laboratory values at the time of hyperkalemia (potassium, creatinine, estimated glomerular filtration rate, white blood cell count, hemoglobin, and platelet), 3-month increase in potassium level, and creatinine ratio (hyperkalemia / 3 months prior). No deaths directly attributable to hyperkalemia were observed in either group. CI: confidence interval; ECG: electrocardiographic; OR: odds ratio.

## Discussion

This retrospective study examined the outcomes of inpatient versus outpatient management in patients presenting with severe hyperkalemia. We found that the management setting was not significantly associated with all-cause mortality; no deaths were directly attributable to hyperkalemia in either group. In contrast, patients managed in the outpatient setting exhibited a higher risk of hyperkalemia recurrence. These findings provide new insights into a long-standing clinical question and may help refine therapeutic strategies for hyperkalemia.

The importance of RAS inhibitors in advanced CKD and the therapeutic benefits of MRAs in diabetic kidney disease have been increasingly recognized in recent years, rendering hyperkalemia associated with these agents a persistent and major clinical issue ^(12), (13)^. Against this backdrop, there is limited evidence on the clinical utility and necessity of hospitalization for patients with severe hyperkalemia. While inpatient care may facilitate intensive potassium monitoring, it also carries significant risks of hospital-acquired infections, functional decline, and delirium, alongside substantial healthcare costs ^(8), (9), (10)^. Therefore, this clinical dilemma is an unresolved issue for optimizing hyperkalemia management. In a pioneering study of 23 patients with severe hyperkalemia (mean K^+^ 6.7 mEq/L), managed either as inpatients (n = 11) or outpatients (n = 12), Charytan et al. ^(7)^ reported no deaths directly attributable to hyperkalemia in either group. The only fatality observed was an inpatient death due to a nosocomial infection. Drawing from these observations, the authors suggested that hospitalization may be unnecessary in the absence of advanced ECG abnormalities, K^+^ ≥8.0 mEq/L, or supervening medical complications. Nevertheless, given the relatively modest sample size and limited clinical granularity of that study, further validation through larger and more comprehensive investigations is warranted.

To address this evidence gap, we analyzed a cohort of more than 500 patients from two tertiary centers in Japan to compare the impact of inpatient versus outpatient management on outcomes among patients with severe hyperkalemia. The overall 90-day mortality was 14%, which appears more modest than previously reported ^(14), (15)^. The observed discrepancy may be explained by the exclusion of patients with CPA upon arrival in this study and the fact that the majority of patients were under regular outpatient management by nephrologists or cardiologists. Furthermore, given the prevalence of advanced CKD and chronically elevated baseline potassium, these patients may have exhibited a degree of physiological tolerance to hyperkalemia ^(16)^. Our weighted logistic regression analysis demonstrated that in patients without acute clinical distress, there was no significant difference in mortality between outpatient and inpatient management. Conversely, the recurrence of hyperkalemia was significantly less frequent in the inpatient group. These results imply that although outpatient care could suffice for acute risk mitigation in selected patients, hospitalization provides a more structured environment for rigorous medication titration and dietary counseling, underscoring a multifaceted role beyond acute stabilization.

This study offers clinically meaningful insights. Over a 90-day period, the comparable mortality indicates that outpatient management—supported by proper risk assessment and medication adjustment—might serve as a viable alternative when hospitalization is infeasible. The introduction of newer potassium-binding agents may further enhance the safety of this strategy ^(17), (18)^. Importantly, the significantly higher recurrence rate in the outpatient group is noteworthy. Hyperkalemia frequently recurs, making its prevention a significant clinical challenge ^(10), (19)^. Taken together, outpatient management may in the long-term contribute to poorer prognosis and increase the burden on healthcare systems. Therefore, although management should be individualized according to patient background and available resources, inpatient care is generally preferable to optimize outcome.

The main strengths of this study lie in its use of detailed clinical data—essential for evaluating the necessity of hospitalization—while securing a larger cohort than previous reports and employing sophisticated statistical methods ^(7)^. However, several limitations should be acknowledged. First, this was an observational study, and residual confounding due to unmeasured factors, such as clinical severity, lifestyle factors, and socioeconomic status, may have influenced both admission decisions and clinical outcomes. In particular, hospitalized patients tended to receive more intensive therapeutic interventions, which may have influenced the observed mortality and recurrence. Causal relationships cannot be established from this study design, although an interventional approach would be practically and ethically infeasible to conduct. Second, our study was conducted in resource-rich urban Japan, and most participants had well-established medical histories. Furthermore, patients with acute clinical instability warranting urgent hospitalization were excluded from the analytic cohort. This selection bias warrants strong caution when extrapolating our findings. Understandably, in settings with limited resources, difficult follow-up, or unclear patient backgrounds, a more conservative management approach remains preferable. Third, the lack of comprehensive ECG data—integral to risk stratification in hyperkalemia—is an important limitation. Although patients with life-threatening arrhythmias were excluded from the main analysis, specific ECG changes such as T-wave amplitude and PR interval could not be considered. Nevertheless, as a significant association between the magnitude of K^+^ elevation and the occurrence of ECG abnormalities has been demonstrated previously, adjustment for this factor in this study may act as a surrogate indicator thereof ^(16)^. Fourth, the observation period was relatively short, at 90 days, and the sample size may be insufficient. Finally, this study was not designed to define explicit criteria for hospitalization in patients with hyperkalemia. Further large-scale prospective observational studies with longer follow-up, building on the findings of this study, are warranted to address this issue and improve the quality of care.

In conclusion, our analysis indicates that among patients with severe hyperkalemia who have no concomitant acute conditions other than the K^+^ elevation itself, outpatient-based care may be feasible without short-term deterioration in survival. Nevertheless, a significantly increased risk of potassium rebound was also demonstrated in this study. Although management decisions depend on the individual clinical scenarios, these results suggest that hospitalization may remain appropriate for patients with severe hyperkalemia to ensure sustained potassium control and patient safety.

## Article Information

### Acknowledgments

The authors thank all participants of this research project.

### Author Contributions

Shinnosuke Sugihara and Takashin Nakayama contributed equally to this work. Shinnosuke Sugihara, Takashin Nakayama, and Tatsuhiko Azegami conceived and designed the study. Shinnosuke Sugihara and Takashin Nakayama performed the statistical analysis and drafted the manuscript. Shinnosuke Sugihara, Takashin Nakayama, and Tatsuhiko Azegami interpreted the data and classified the primary outcome. Motoaki Komatsu and Kaori Hayashi supervised the entire study. All authors critically reviewed and revised the manuscript and approved the final version for submission.

### Conflicts of Interest

None

### Ethics Approval

The study protocol was approved by the Ethics Committee of Keio University School of Medicine (approval number: 20251164).

### Data Availability

The data underlying this article will be shared on reasonable request to the corresponding author.

## Supplement

Supplementary Material
