# Supplementary material for: Clinical Outcomes of Hospitalization for Severe Hyperkalemia in the Absence of Life-threatening Manifestations
Source: JMA J. 2026 Aug 21;9(5):1263–70. doi: 10.31662/jmaj.2026-0089 (PMC13639416; doi:10.31662/jmaj.2026-0089)
Supplement: Supplementary file 1 [file 2433-3298-9-5-1263-s001.pdf]

Table S1. Characteristics of Patients with Severe Hyperkalemia in the Absence of Life-Threatening ECG Abnormalities or Concurrent Acute Conditions: before and after Overlap Weighting.

	Before weighting			After weighting		
	Inpatient (n = 99)	Outpatient (n = 275)	Absolute SMD	Inpatient	Outpatient	absolute SMD
Age, years	75 (68–83)	74 (65–81)	0.14	75	75	<0.01
Male	65 (66%)	208 (76%)	0.22	0.71	0.71	<0.01
Body mass index, kg/m²	21.8 (19.5–25.3)	23.3 (20.6–26.1)	0.2	23	23	<0.01
Do-not-resuscitate code	14 (14%)	12 (4%)	0.34	0.07	0.07	<0.01
Visit pattern						
Scheduled	56 (57%)	270 (98%)	1.14	0.92	0.92	<0.01
Unscheduled	43 (43%)	5 (2%)		0.08	0.08	
Comorbidities						
Diabetes mellitus	45 (45%)	157 (57%)	0.23	0.53	0.53	<0.01
Heart failure	32 (32%)	97 (35%)	0.06	0.35	0.35	<0.01
Coronary artery disease	19 (19%)	61 (22%)	0.07	0.19	0.19	<0.01
Stroke	9 (9%)	41 (15%)	0.18	0.12	0.12	<0.01
Malignancy	45 (45%)	94 (34%)	0.23	0.4	0.4	<0.01
Concomitant medications						
RAS inhibitors	70 (71%)	203 (74%)	0.07	0.78	0.78	<0.01
MRA	26 (26%)	64 (23%)	0.07	0.28	0.28	<0.01
Beta-blockers	34 (34%)	77 (28%)	0.14	0.35	0.35	<0.01
Loop diuretics	31 (31%)	77 (28%)	0.07	0.35	0.35	<0.01
Bicarbonate	7 (7%)	33 (12%)	0.17	0.09	0.09	<0.01
Potassium binders	12 (12%)	59 (21%)	0.25	0.18	0.18	<0.01
Laboratory values						
At hyperkalemia						
K^+, mEq/L	6.8 (6.6–7.1)	6.6 (6.5–6.8)	0.69	6.8	6.8	<0.01

Creatinine, mg/dL	2.6 (1.8–3.8)	2.5 (1.7–4.2)	0.15	3.1	3.1	<0.01
eGFR, mL/min/1.73 m²	18 (12–27)	20 (12–30)	0.17	22	22	<0.01
WBC, ×10⁹/L	7.2 (5.8–9.6)	6.4 (5.1–7.7)	0.43	7.1	7.1	<0.01
Hemoglobin, g/dL	11.1 (9.6–13.0)	10.9 (9.8–12.3)	0.06	10.8	10.8	<0.01
Platelet, ×10⁹/L	211 (167–301)	195 (153–243)	0.39	219	219	<0.01
3-month increase in K^+, mEq/L	2.1 (1.4–2.6)	1.2 (0.9–1.7)	0.93	1.7	1.7	<0.01
Creatinine ratio (hyperkalemia / 3 months prior)	1.4 (1.1–2.2)	1.1 (1.0–1.2)	0.53	1.4	1.4	<0.01

Note: Data before weighting are presented as median (interquartile range) or n (%). Values after overlap weighting are weighted means for continuous variables and weighted proportions for categorical variables.

ECG: electrocardiographic; eGFR: estimated glomerular filtration rate; K^+: serum potassium level; MRA: mineralocorticoid receptor antagonist; RAS: renin–angiotensin system; SMD: standardized mean difference; WBC: white blood cell count.
